# Supplementary material for: Hyak mortality monitoring system: innovative sampling and estimation methods – proof of concept by simulation
Source: Glob Health Epidemiol Genom. 2018 Feb 5;3:e3. doi: 10.1017/gheg.2017.15 (PMC5870438; doi:10.1017/gheg.2017.15)
Supplement: Supplementary file 1 [file S205442001700015Xsup001.pdf]

A Appendix

A.1 Optimum allocation sampling strategy details

Suppose we have stratum, indexed by $i = 1, \ldots, I$. In our case the strata are areas. Let N_i be the population of area i and $N = \sum_i N_i$ the total population in the study region.

Let $Y_{ik} = 0/1$ be the indicator of whether child k in area i died, $k = 1, \ldots, N_i$, $i = 1, \ldots, I$. Then we are interested in $T = \sum_i \sum_k Y_{ik}$, the total number of deaths. The fraction of deaths is $\overline{y} = \widehat{p} = T/N$.

Let $q_i = N_i/N$ and S_i be the standard deviation of the response in stratum i where

$$S_i^2 = \frac{N_i}{N_i - 1} p_i(1 - p_i) \approx p_i(1 - p_i),$$

which is estimated by

$$s_i^2 = \frac{n_i}{n_i - 1} \widehat{p}_i(1 - \widehat{p}_i) \approx \widehat{p}_i(1 - \widehat{p}_i).$$

If we use the usual estimator of $\widehat{p}_i = \sum_{k=1}^{n_i} y_{ik}/n_i$ then the variance is

$$var(\overline{y}) = \sum_{i=1}^{I} q_i^2 (1 - f_i) \frac{S_i^2}{n_i} = \sum_{i=1}^{I} q_i^2 (1 - f_i) \frac{N_i}{N_i - 1} \frac{p_i(1 - p_i)}{n_i},$$

where $f_i = n_i/N_i$, which leads to

$$var(\widehat{T}) = N^2 \sum_{i=1}^{I} q_i^2 (1 - f_i) \frac{S_i^2}{n_i} = N^2 \sum_{i=1}^{I} q_i^2 (1 - f_i) \frac{p_i(1 - p_i)}{n_i - 1}.$$

Substituting in $\widehat{p}_i$ gives the estimated variances.

We wish to choose n_i, the number of samples to take in area i.

Then the optimum allocation, in the sense of minimizing $var(\overline{y})$ (which is the same as minimizing the variance of T) is Neyman allocation (Lohr, 2010) in which

$$n_i = n \frac{q_i S_i}{\sum_i q_i S_i}. \tag{5}$$

Note: we really should be minimizing MSE as our estimators are biased (since they are random effects models with shrinkage).

In our setting, we have an estimate of p_i and so we can use this in (5) which becomes

$$n_i \approx n \times \frac{q_i \sqrt{\widehat{p}_i(1 - \widehat{p}_i)}}{\sum_{i'} q_{i'} \sqrt{\widehat{p}_{i'}(1 - \widehat{p}_{i'})}}. \tag{6}$$

We do not include the age-gender groups j in our sampling strata, but our model produces estimates $\widehat{p}_{ij}$ so we estimate $\widehat{p}_i$ via

$$\widehat{p}_i = \sum_{j=1}^{J} \frac{N_{ij}}{N_i} \widehat{p}_{ij},$$

to use in (6).

A.2 Village-level characteristics for the current and historic cohorts

Tables A.1 and A.2 display the village characteristics for both the current-day and historical cohorts. The current-day cohort is the fixed population from which we draw repeated samples, while the historical cohort is used by the Hyak and optimum sampling schemes to obtain estimated village-level probabilities of death. In our simulation, we used villages 4, 7 and 8 as the HDSS sites.

Table A.1: Village characteristics for current-day cohort. This cohort represents our fixed population from which we draw repeated samples.

Village	Number of Households	Number of Children	# Deaths	P(Death)	$\boldsymbol{x}_1$	$\boldsymbol{x}_2$
1	4221	12523	1654	0.13	0.56	0.70
2	1376	4150	119	0.03	0.92	0.32
3	3050	9172	169	0.02	0.89	0.55
4	3804	11331	483	0.04	0.92	0.56
5	1275	3802	492	0.13	0.39	0.68
6	1515	4550	156	0.03	0.58	0.17
7	3036	9011	929	0.10	0.77	0.98
8	2648	7870	554	0.07	0.32	0.07
9	1957	5841	658	0.11	0.55	0.83
10	3532	10630	500	0.05	0.57	0.47
11	2679	7981	1286	0.16	0.10	0.60
12	2034	6043	413	0.07	0.05	0.83
13	2082	6291	218	0.03	0.73	0.17
14	3320	9901	939	0.09	0.76	0.96
15	2466	7361	196	0.03	0.53	0.51
16	2467	7301	531	0.07	0.66	0.44
17	709	2092	230	0.11	0.04	0.51
18	1192	3610	725	0.20	0.02	0.76
19	3083	9300	600	0.06	0.62	0.27
20	836	2482	447	0.18	0.09	0.97

Table A.2: Village characteristics for historical cohort. The HDSS villages are 4, 7 and 8.

Village	Number of Households	Number of Children	# Deaths	P(Death)	$\boldsymbol{x}_1$	$\boldsymbol{x}_2$
1	1460	4331	587	0.14	0.56	0.70
2	4064	12001	331	0.03	0.92	0.32
3	524	1552	33	0.02	0.89	0.55
4	2927	8720	377	0.04	0.92	0.56
5	4022	11891	1499	0.13	0.39	0.68
6	4157	12450	393	0.03	0.58	0.17
7	2873	8532	919	0.11	0.77	0.98
8	1529	4540	322	0.07	0.32	0.07
9	4108	12152	1292	0.11	0.55	0.83
10	1570	4640	231	0.05	0.57	0.47
11	2789	8342	1444	0.17	0.10	0.60
12	3685	10931	693	0.06	0.05	0.83
13	1786	5242	165	0.03	0.73	0.17
14	674	2070	187	0.09	0.76	0.96
15	473	1402	31	0.02	0.53	0.51
16	3187	9550	735	0.08	0.66	0.44
17	4344	13080	1329	0.10	0.04	0.51
18	3449	10302	2058	0.20	0.02	0.76
19	3080	9191	666	0.07	0.62	0.27
20	468	1422	286	0.20	0.09	0.97

A.3 Additional simulation results

Tables A.3, A.4, and A.5 summarize the results of the simulation study for $n = 3,900, n = 2,600$ and $n = 1,300$, respectively. The number of average sampled deaths and bias, variance and MSE from (4) are displayed for each combination of sampling strategy and analytical model.

Table A.3: Deaths, Bias, Variance, MSE for cluster sampling, stratified sampling, HYAK and optimum sampling for $n = 3,900$. Results from $S = 100$ simulations. There were 11,299 deaths in the simulated population from which samples were taken. 'Cluster' is shorthand for *Two-stage Cluster Sample*; 'HYAK' for *HDSS with Informative Sampling*; 'Strata/Covariates' for *Logistic Regression Covariate Model* and 'Strata/Covariates/Space' for *Logistic Regression Random Effects Covariate Model*. It is not possible to fit the spatial model (IV) to the two-stage cluster sampling scheme since there are data from 5 villages only.

Design	Model	Deaths	Bias	Variance ($\times 10^3$)	MSE ($\times 10^3$)
Cluster	I. Naïve	342	1,072	192	1,342
	II. Strata	342	878	207	977
	III. Strata/Covariates	342	644	775	1,190
	IV. Strata/Covariates/Space	342	—	—	—
Stratified	I. Naïve	344	1,066	9	1,145
	II. Strata	344	871	26	785
	III. Strata/Covariates	344	660	25	460
	IV. Strata/Covariates/Space	344	225	99	150
Hyak	I. Naïve	409	1,181	8	1,402
	II. Strata	409	982	25	988
	III. Strata/Covariates	409	640	22	431
	IV. Strata/Covariates/Space	409	188	92	128
Optimum	I. Naïve	356	1,079	7	1,171
	II. Strata	356	885	23	806
	III. Strata/Covariates	356	642	23	436
	IV. Strata/Covariates/Space	356	194	85	123

Table A.4: Deaths, Bias, Variance, MSE for cluster sampling, stratified sampling, HYAK and optimum sampling for $n = 2,600$. Results from $S = 100$ simulations. There were 11,299 deaths in the simulated population from which samples were taken. 'Cluster' is shorthand for *Two-stage Cluster Sample*; 'HYAK' for *HDSS with Informative Sampling*; 'Strata/Covariates' for *Logistic Regression Covariate Model* and 'Strata/Covariates/Space' for *Logistic Regression Random Effects Covariate Model*. It is not possible to fit the spatial model (IV) to the two-stage cluster sampling scheme since there are data from 5 villages only.

Design	Model	Deaths	Bias	Variance ($\times 10^3$)	MSE ($\times 10^3$)
Cluster	I. Naïve	250	1,075	170	1,326
	II. Strata	250	881	190	966
	III. Strata/Covariates	250	659	382	816
	IV. Strata/Covariates/Space	250	—	—	—
Stratified	I. Naïve	256	1,075	11	1,166
	II. Strata	256	879	30	802
	III. Strata/Covariates	256	664	27	468
	IV. Strata/Covariates/Space	256	248	123	185
Hyak	I. Naïve	302	1,193	15	1,439
	II. Strata	302	992	41	1,025
	III. Strata/Covariates	302	646	30	448
	IV. Strata/Covariates/Space	302	209	109	152
Optimum	I. Naïve	264	1,090	10	1,198
	II. Strata	264	893	31	829
	III. Strata/Covariates	264	646	29	446
	IV. Strata/Covariates/Space	264	223	109	159

Table A.5: Deaths, Bias, Variance, MSE for cluster sampling, stratified sampling, HYAK and optimum sampling for $n = 1,300$. Results from $S = 100$ simulations. There were 11,299 deaths in the simulated population from which samples were taken. 'Cluster' is shorthand for *Two-stage Cluster Sample*; 'HYAK' for *HDSS with Informative Sampling*; 'Strata/Covariates' for *Logistic Regression Covariate Model* and 'Strata/Covariates/Space' for *Logistic Regression Random Effects Covariate Model*. It is not possible to fit the spatial model (IV) to the two-stage cluster sampling scheme since there are data from 5 villages only.

Design	Model	Deaths	Bias	Variance ($\times 10^3$)	MSE ($\times 10^3$)
Cluster	I. Naïve	113	1,079	193	1,358
	II. Strata	113	886	241	1,025
	III. Strata/Covariates	113	662	1,252	1,690
	IV. Strata/Covariates/Space	113	—	—	—
Stratified	I. Naïve	119	1,088	23	1,205
	II. Strata	119	895	62	863
	III. Strata/Covariates	119	662	60	499
	IV. Strata/Covariates/Space	119	325	196	301
Hyak	I. Naïve	138	1,193	24	1,447
	II. Strata	138	1,001	70	1,071
	III. Strata/Covariates	138	655	61	491
	IV. Strata/Covariates/Space	138	309	175	271
Optimum	I. Naïve	122	1,100	27	1,238
	II. Strata	122	902	78	891
	III. Strata/Covariates	122	658	68	500
	IV. Strata/Covariates/Space	122	306	203	297

Figures A.1-A.3 display the distributions of the estimated probability of dying produced by each model (models I, III & IV – *Naïve, Covariates* and *Covariates & Space*) under the Hyak sampling strategy for $n = 3,900, n = 2,600$ and $n = 1,300$, respectively.

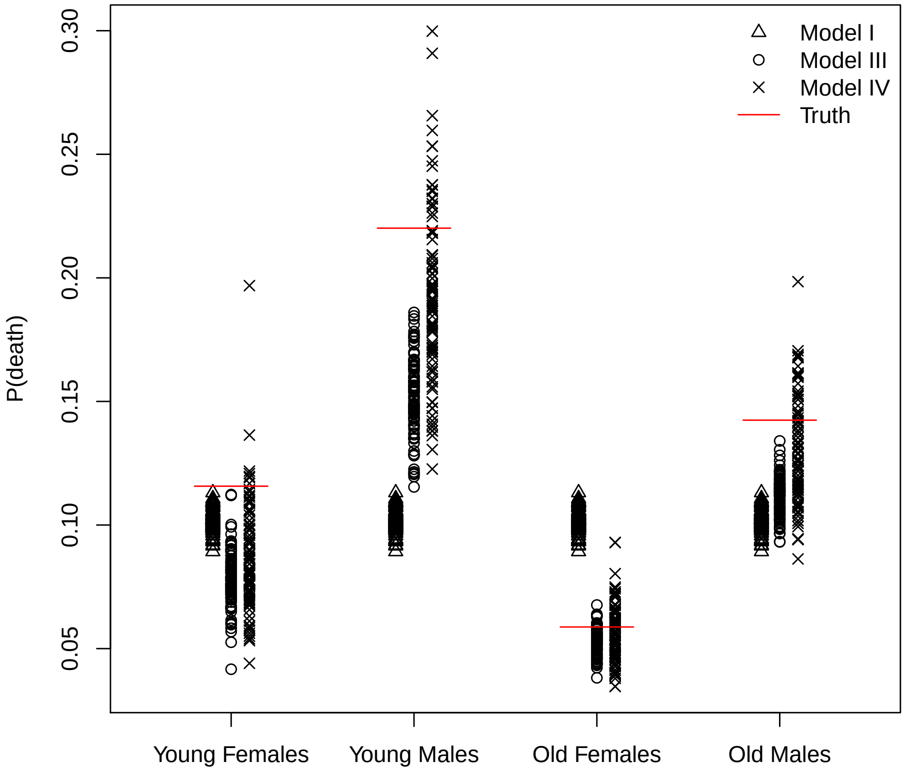

Figure A.1: The distributions of the estimated probability of dying from models I, III and IV under the Hyak sampling strategy for $n = 3,900$.

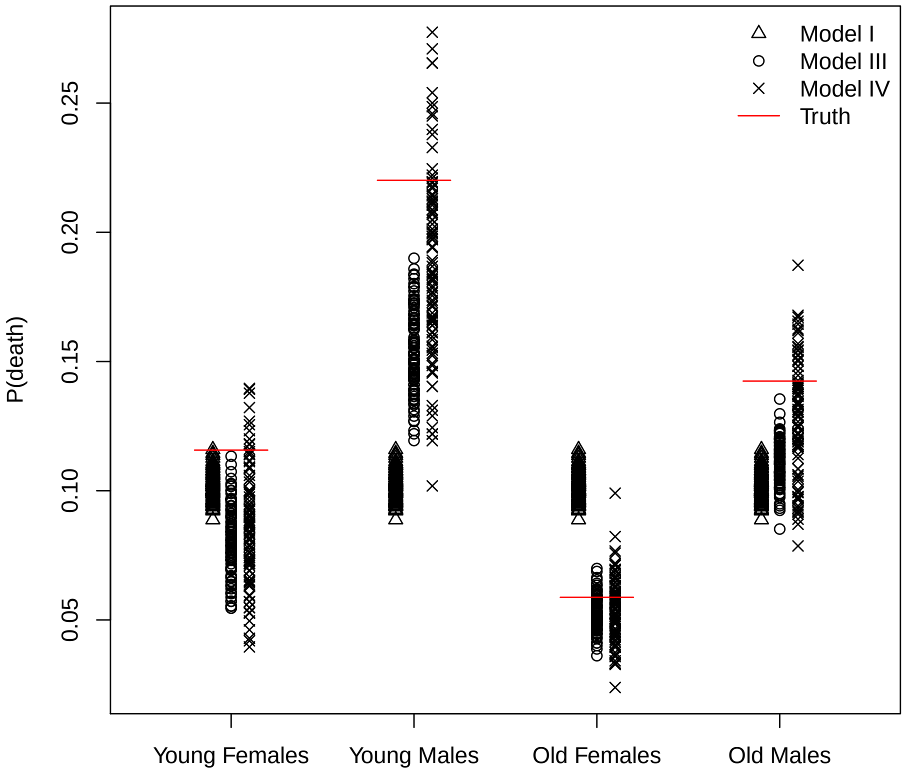

Figure A.2: The distributions of the estimated probability of dying from models I, III and IV under the HYAK sampling strategy for $n = 2,600$.

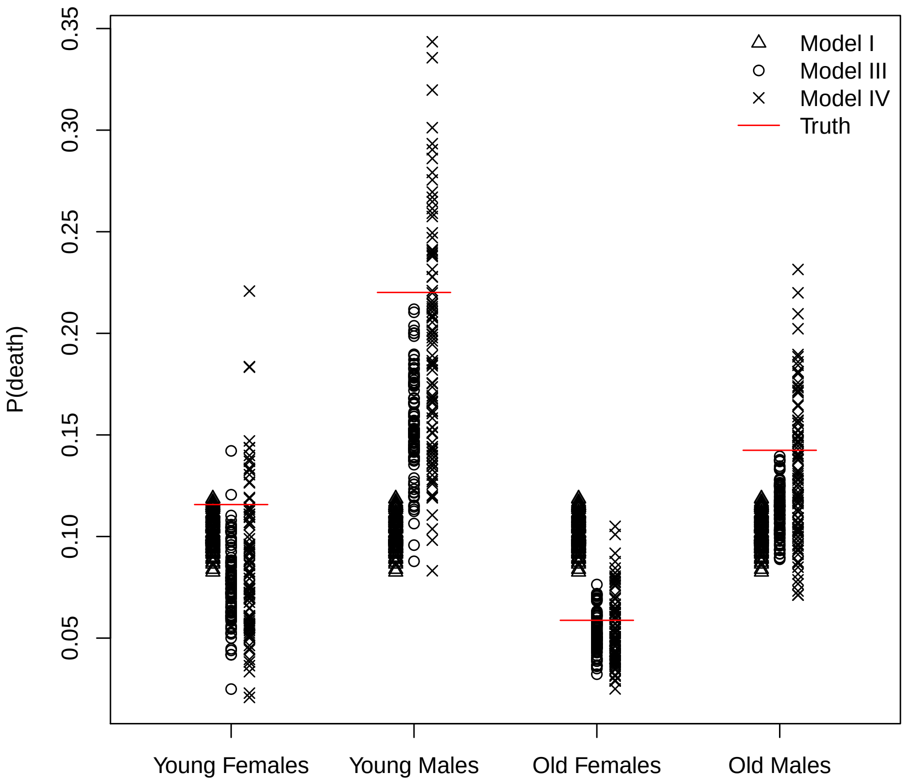

Figure A.3: The distributions of the estimated probability of dying from models I, III and IV under the HYAK sampling strategy for $n = 1,300$.

Figures A.4-A.6 display the average village- and strata-specific estimates for the (unobserved) population counts of death plotted against the true values across each of the four models under the Hyak sampling scheme for $n = 3,900, n = 2,600$ and $n = 1,300$, respectively.

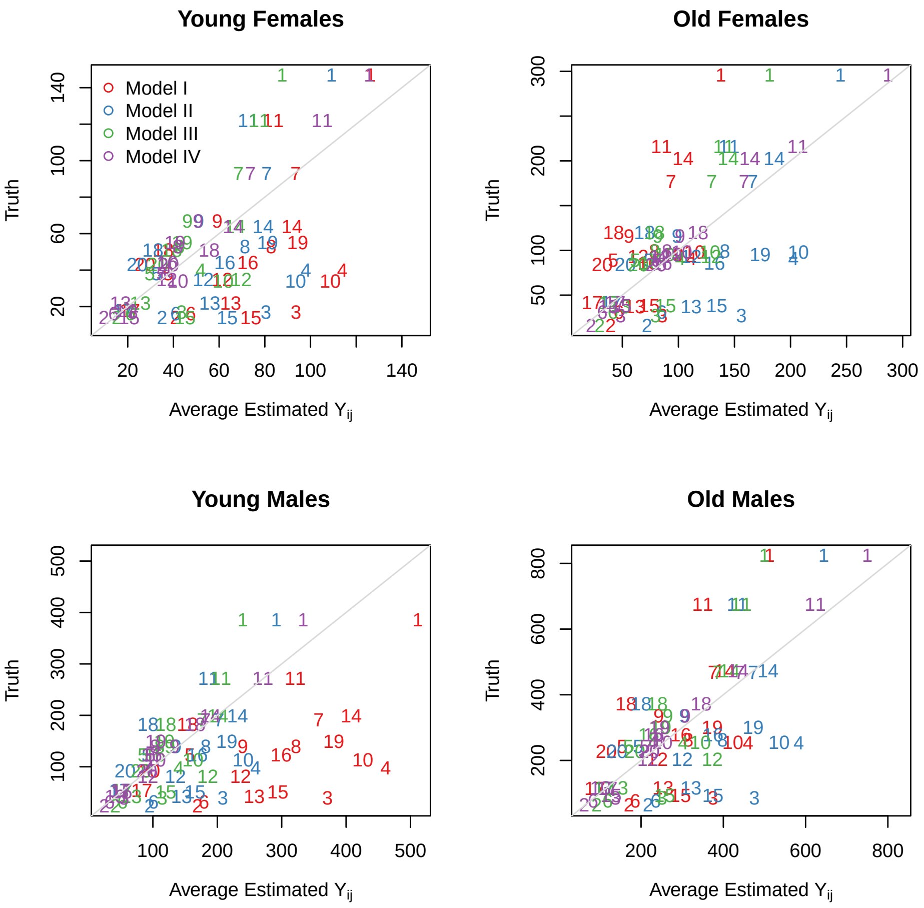

Figure A.4: The average village- and strata-specific estimates for the (unobserved) population counts of death plotted against the true values across each of the four models under the Hyak sampling scheme for $n = 3,900$. Plotting symbols indicate village numbers.

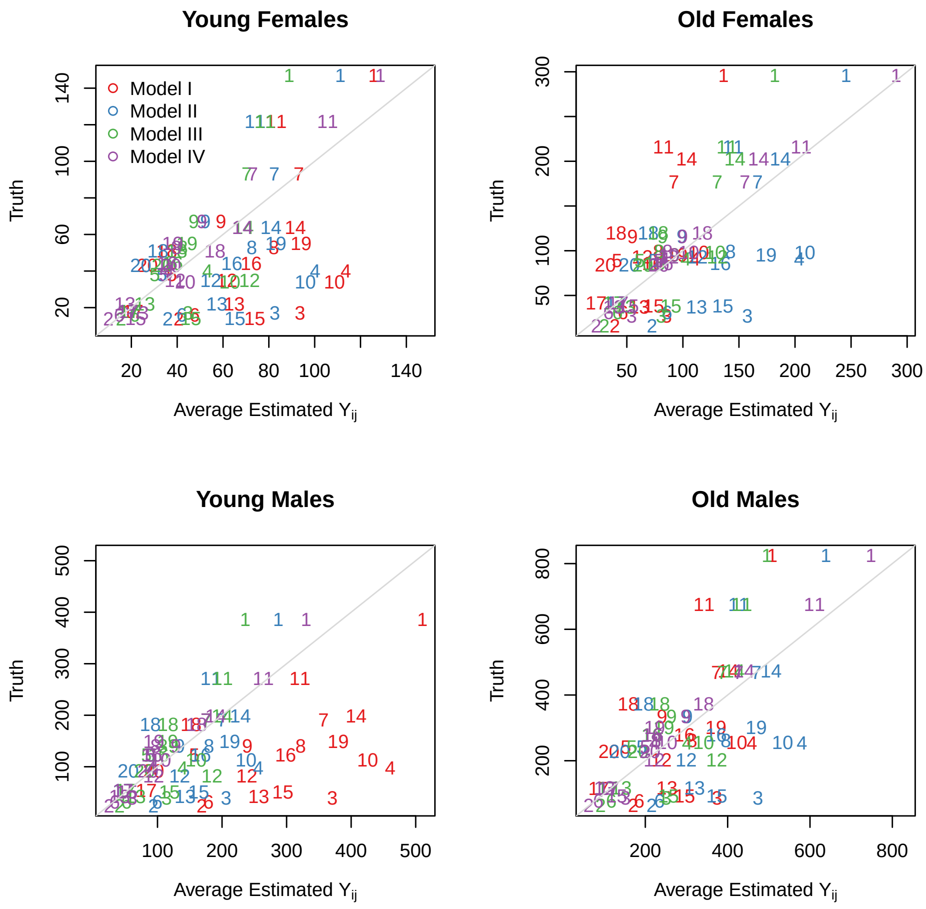

Figure A.5: The average village- and strata-specific estimates for the (unobserved) population counts of death plotted against the true values across each of the four models under the HYAK sampling scheme for $n = 2,600$. Plotting symbols indicate village numbers.

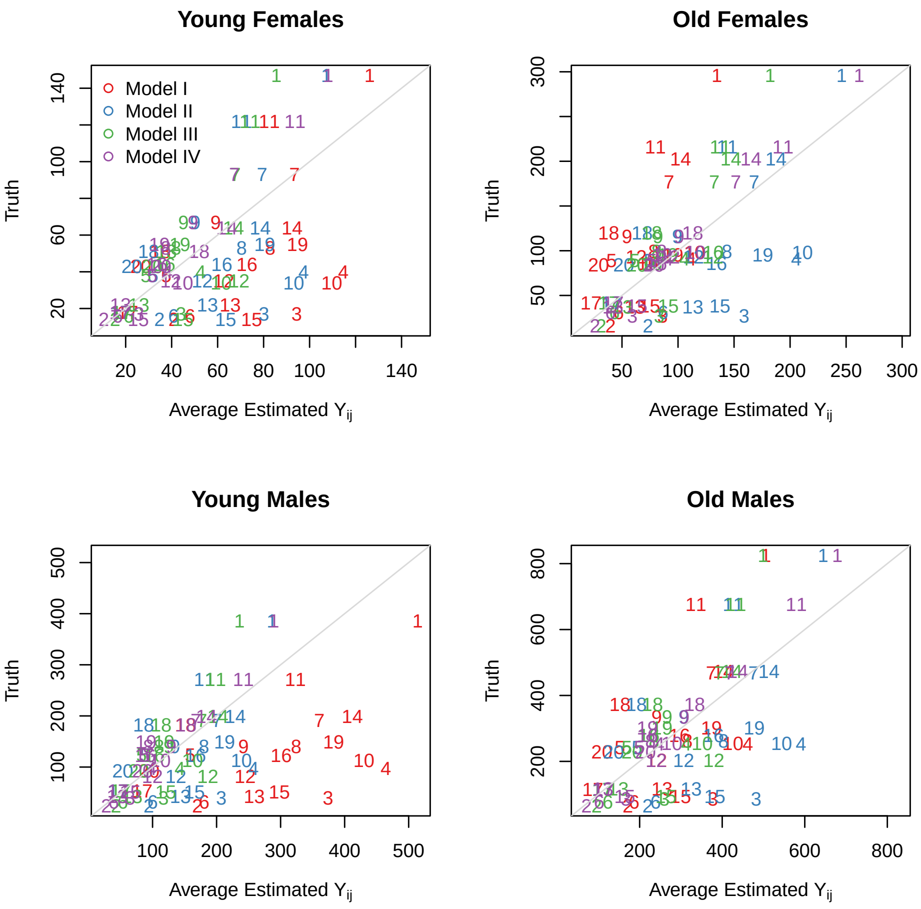

Figure A.6: The average village- and strata-specific estimates for the (unobserved) population counts of death plotted against the true values across each of the four models under the HYAK sampling scheme for $n = 1,300$. Plotting symbols indicate village numbers.

Figures A.7-A.10 display the average village- and strata-specific estimates for the (unobserved) population counts of death plotted against the true values across each of the four models under the two-stage cluster sampling scheme for $n = 5,200, n = 3,900, n = 2,600$ and $n = 1,300$, respectively.

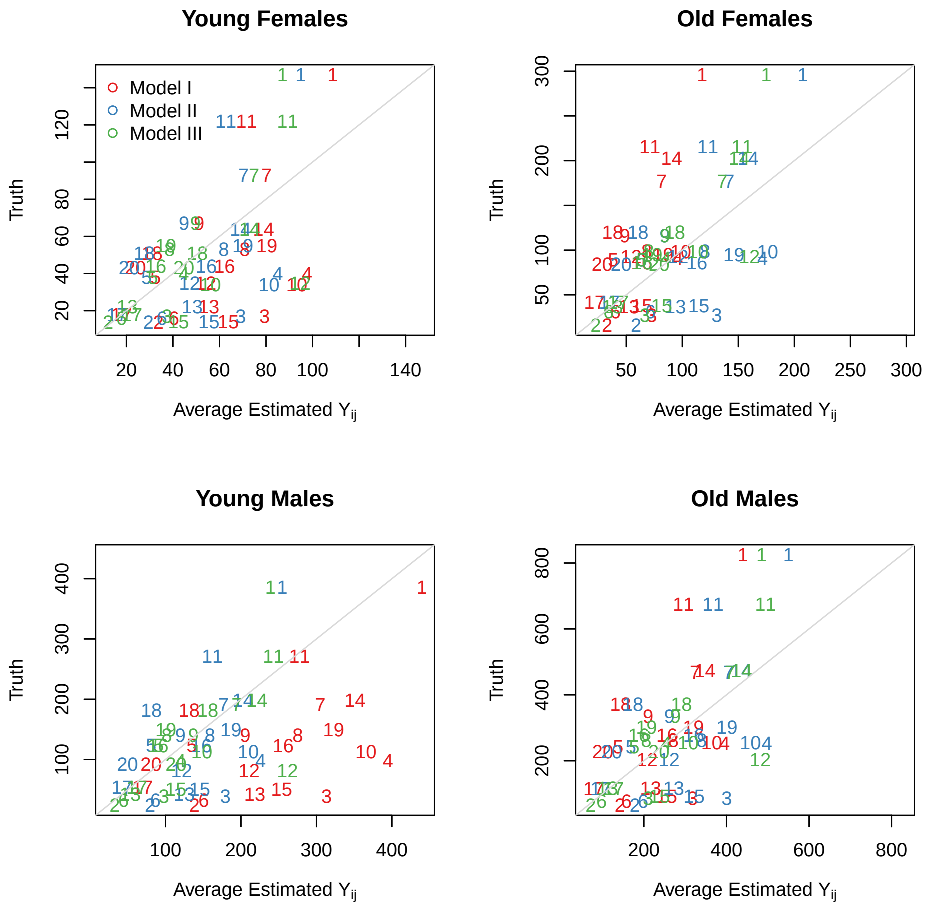

Figure A.7: The average village- and strata-specific estimates for the (unobserved) population counts of death plotted against the true values across each of the four models under the two-stage cluster sampling scheme for $n = 5,200$. Plotting symbols indicate village numbers.

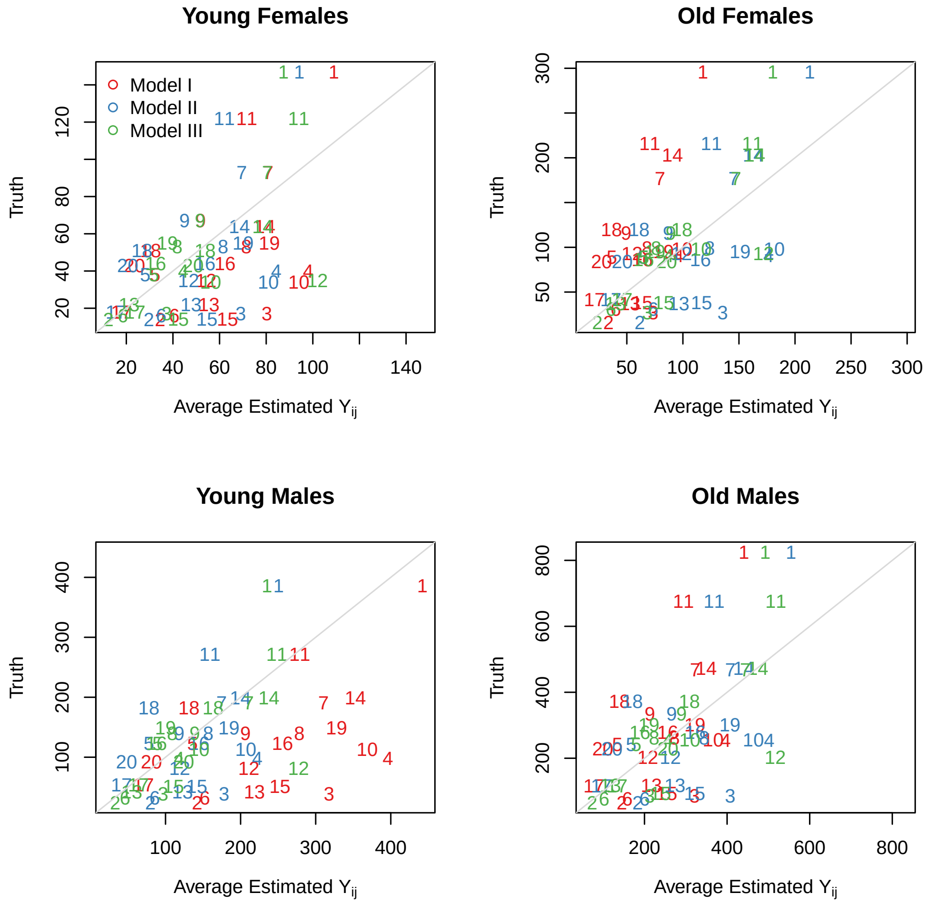

Figure A.8: The average village- and strata-specific estimates for the (unobserved) population counts of death plotted against the true values across each of the four models under the two-stage cluster sampling scheme for $n = 3,900$. Plotting symbols indicate village numbers.

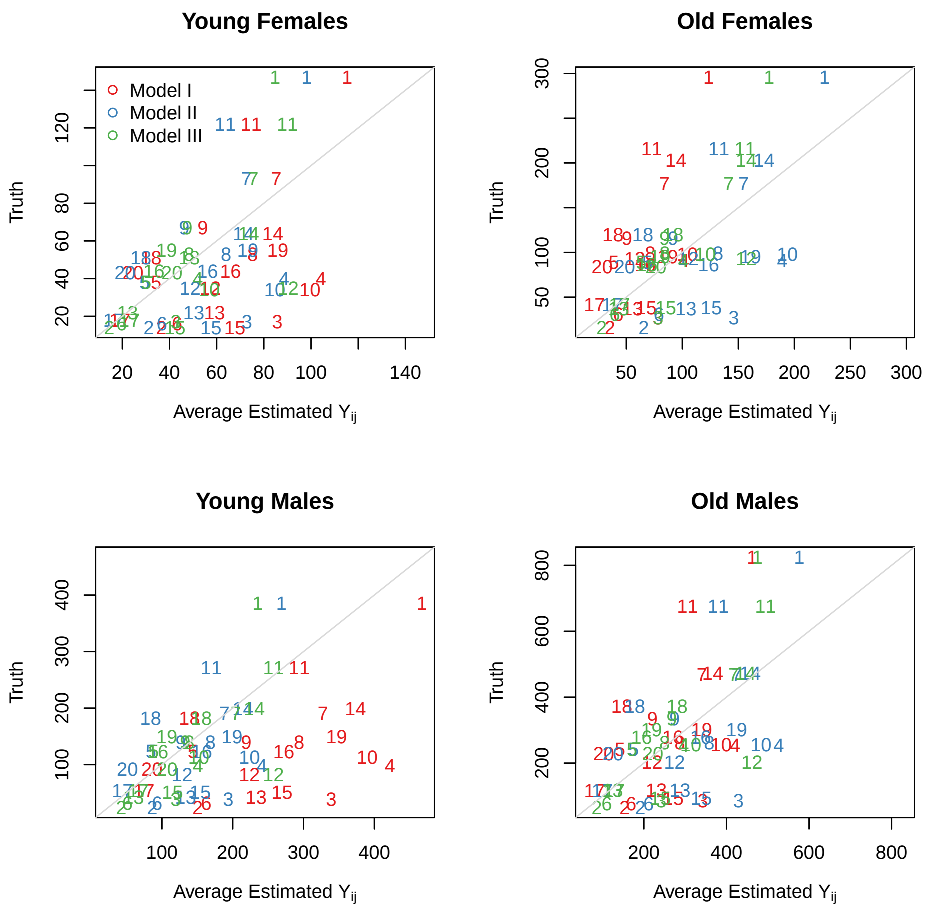

Figure A.9: The average village- and strata-specific estimates for the (unobserved) population counts of death plotted against the true values across each of the four models under the two-stage cluster sampling scheme for $n = 2,600$. Plotting symbols indicate village numbers.

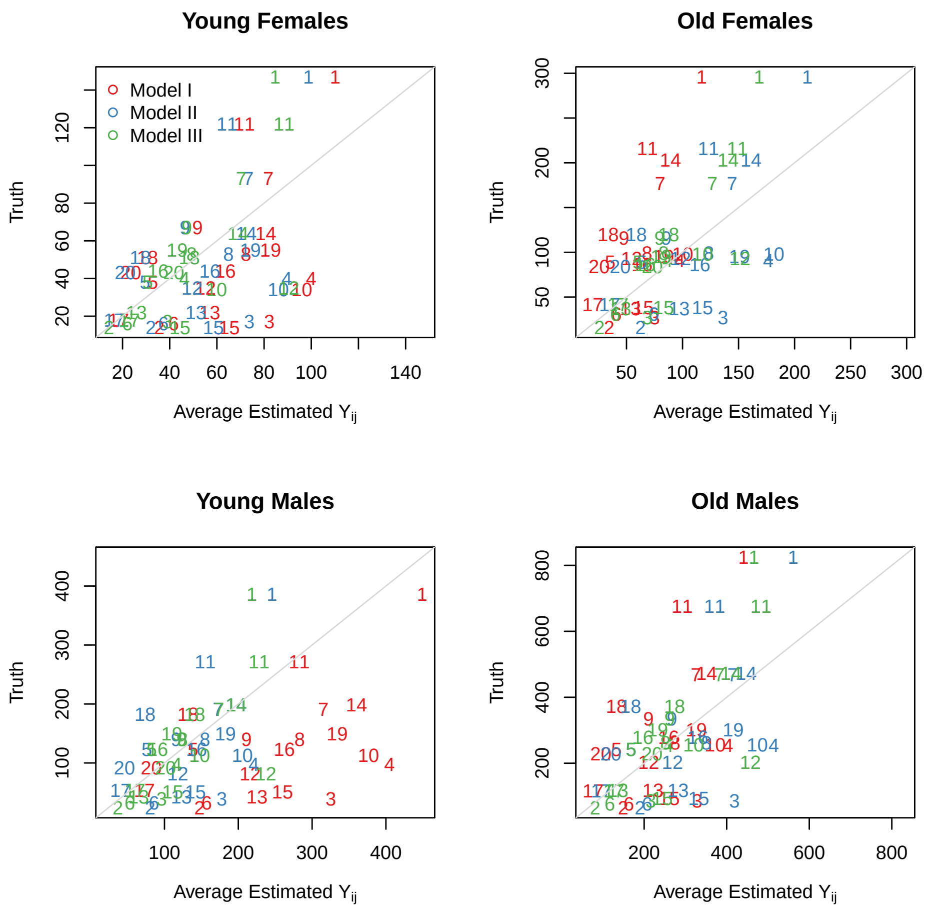

Figure A.10: The average village- and strata-specific estimates for the (unobserved) population counts of death plotted against the true values across each of the four models under the two-stage cluster sampling scheme for $n = 1,300$. Plotting symbols indicate village numbers.

Figures A.11-A.14 display the average village- and strata-specific estimates for the (unobserved) population counts of death plotted against the true values across each of the four models under the simple random sampling scheme for $n = 5,200, n = 3,900, n = 2,600$ and $n = 1,300$, respectively.

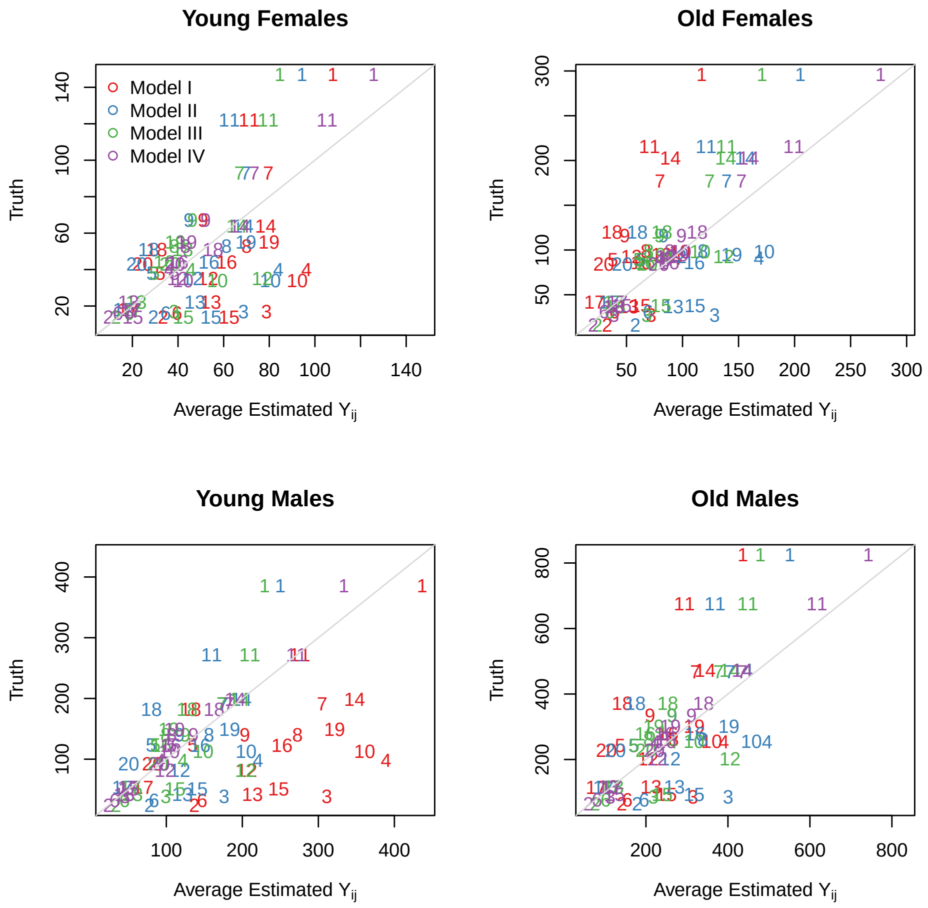

Figure A.11: The average village- and strata-specific estimates for the (unobserved) population counts of death plotted against the true values across each of the four models under the simple random sampling scheme for $n = 5,200$. Plotting symbols indicate village numbers.

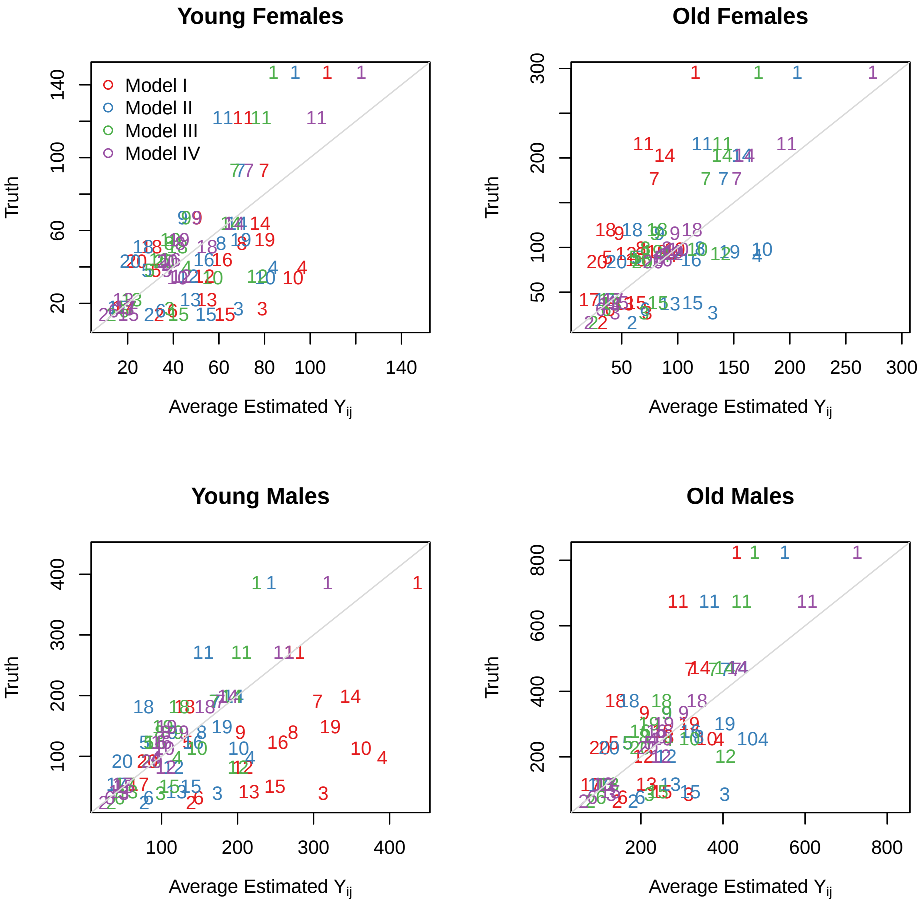

Figure A.12: The average village- and strata-specific estimates for the (unobserved) population counts of death plotted against the true values across each of the four models under the simple random sampling scheme for $n = 3,900$. Plotting symbols indicate village numbers.

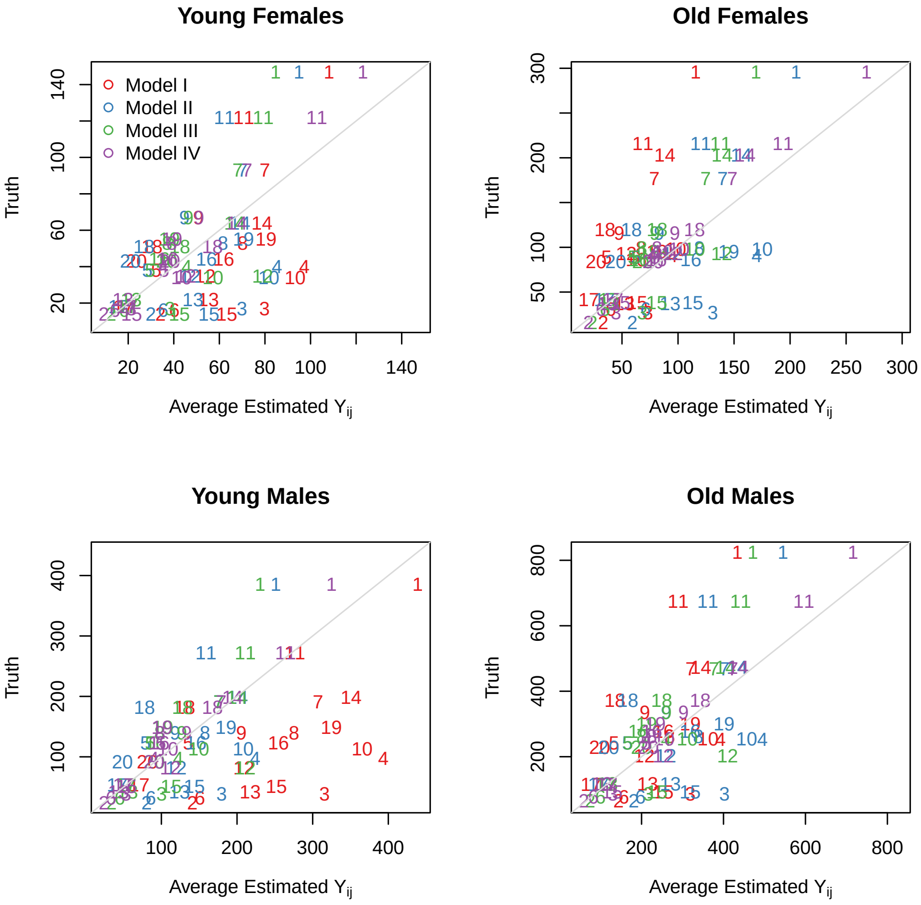

Figure A.13: The average village- and strata-specific estimates for the (unobserved) population counts of death plotted against the true values across each of the four models under the simple random sampling scheme for $n = 2,600$. Plotting symbols indicate village numbers.

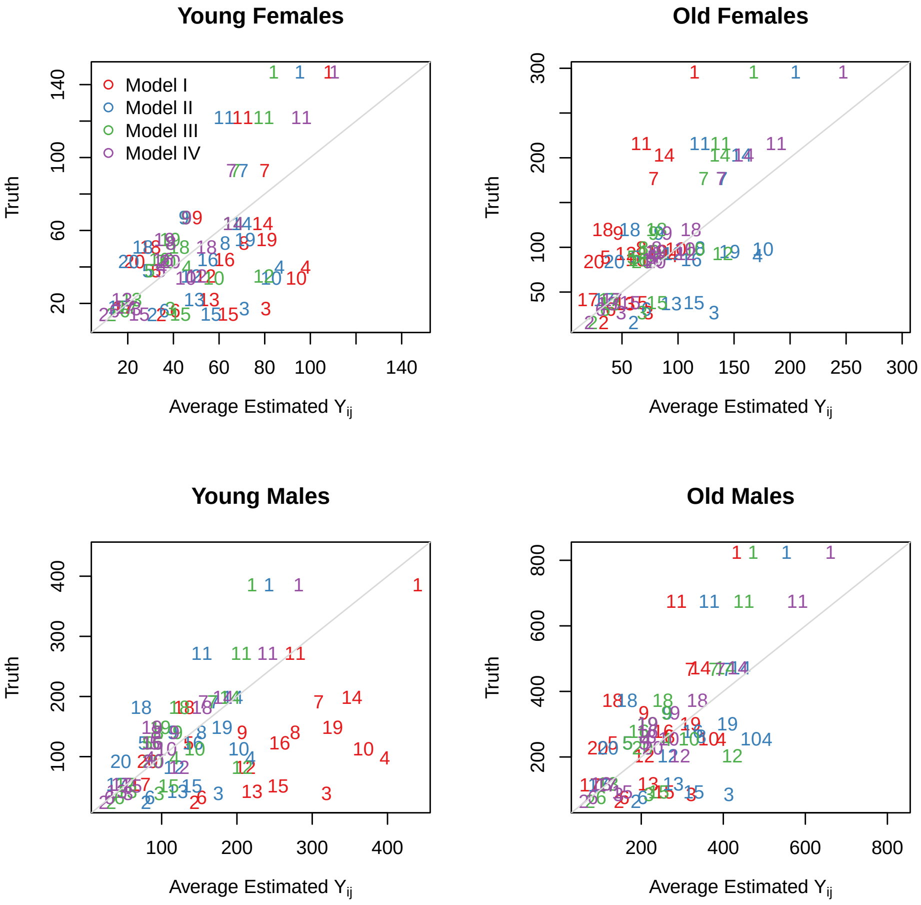

Figure A.14: The average village- and strata-specific estimates for the (unobserved) population counts of death plotted against the true values across each of the four models under the simple random sampling scheme for $n = 1,300$. Plotting symbols indicate village numbers.

Figures A.15-A.18 display the average village- and strata-specific estimates for the (unobserved) population counts of death plotted against the true values across each of the four models under the optimum sampling scheme for $n = 5,200$, $n = 3,900$, $n = 2,600$ and $n = 1,300$, respectively.

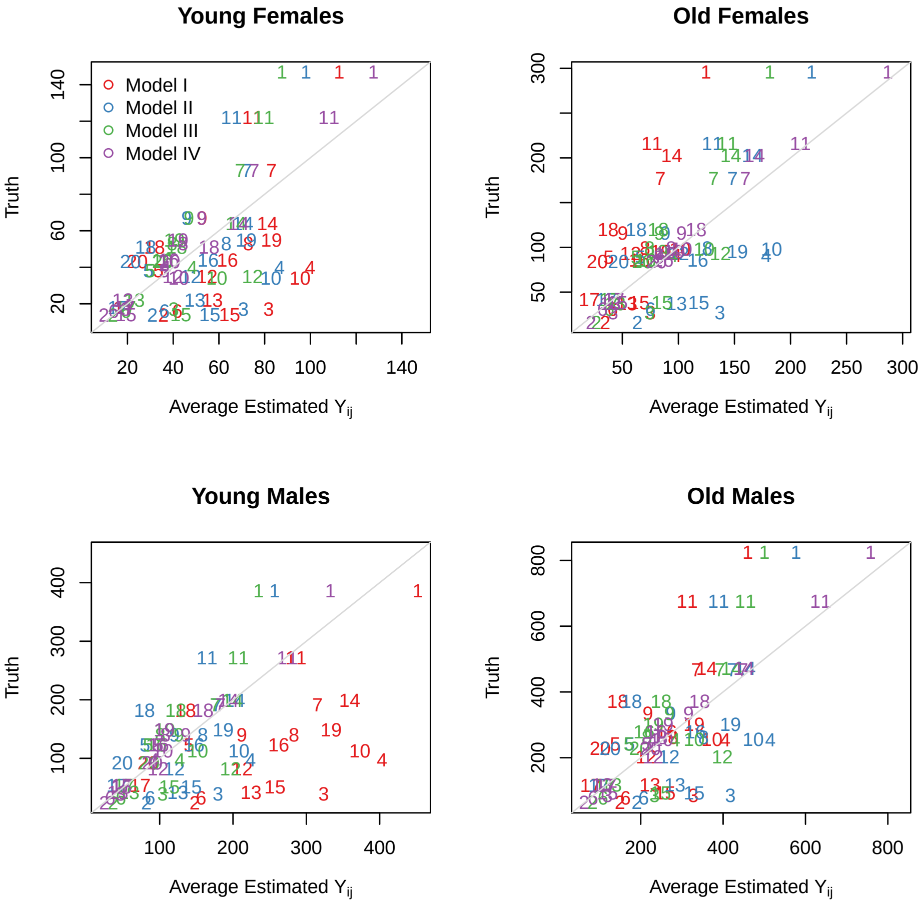

Figure A.15: The average village- and strata-specific estimates for the (unobserved) population counts of death plotted against the true values across each of the four models under the optimum sampling scheme for $n = 5,200$. Plotting symbols indicate village numbers.

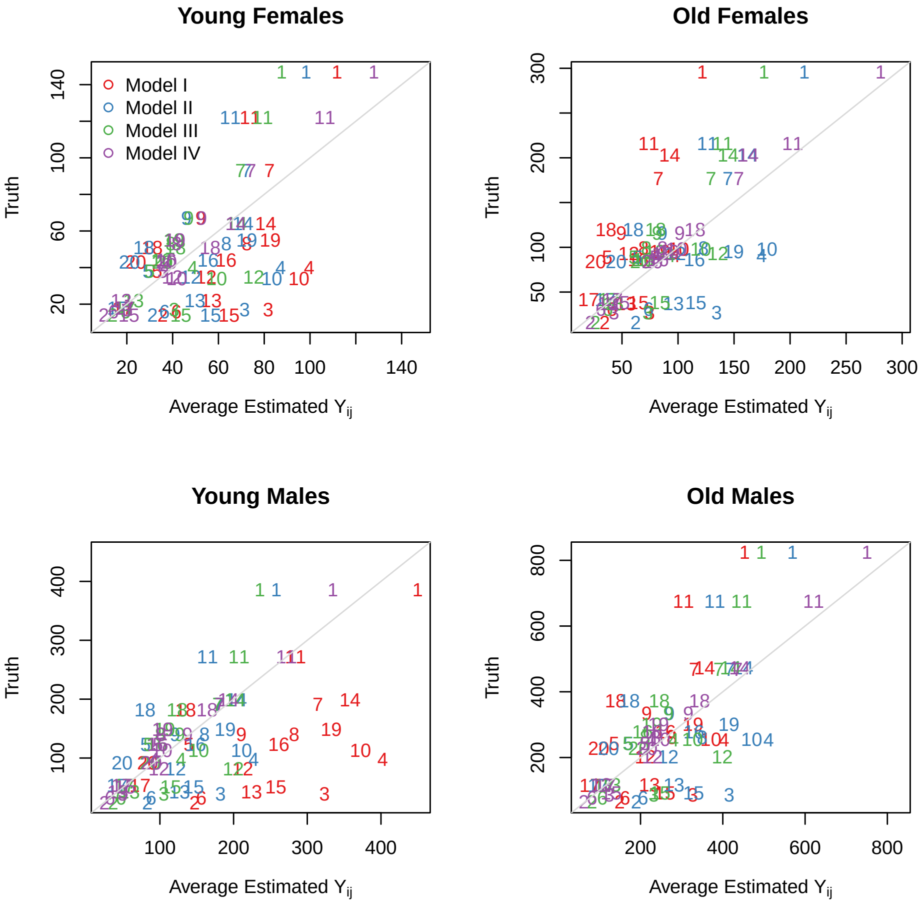

Figure A.16: The average village- and strata-specific estimates for the (unobserved) population counts of death plotted against the true values across each of the four models under the optimum sampling scheme for $n = 3,900$. Plotting symbols indicate village numbers.

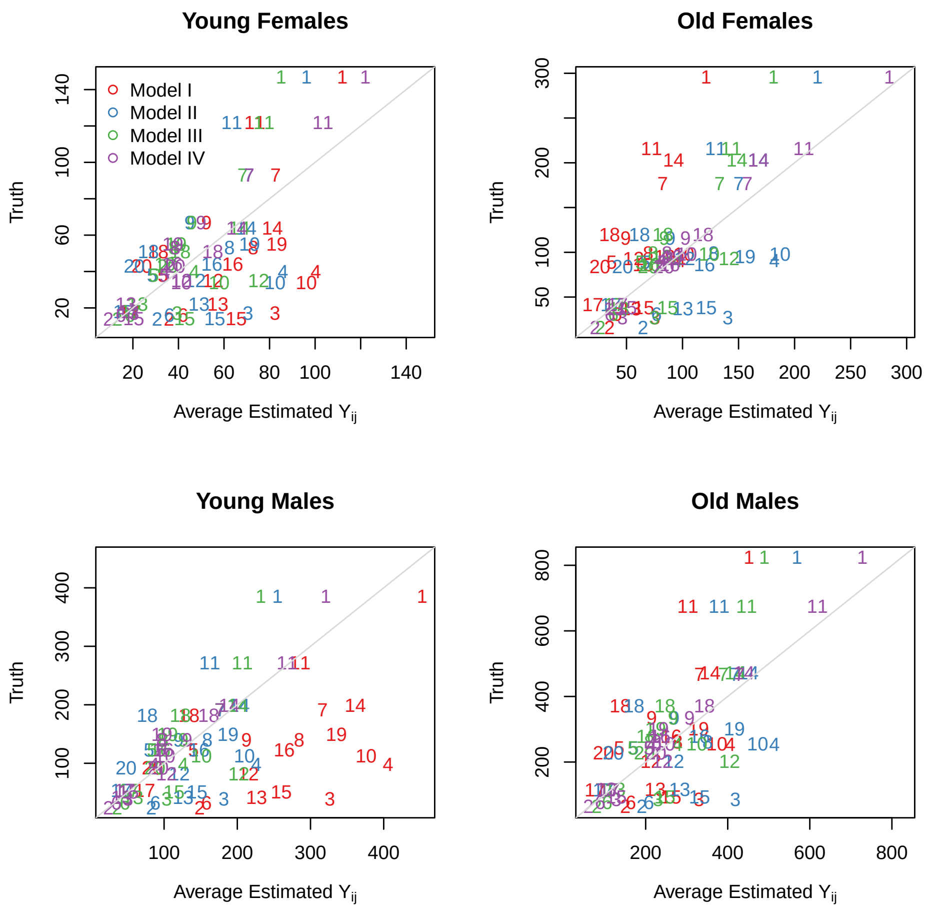

Figure A.17: The average village- and strata-specific estimates for the (unobserved) population counts of death plotted against the true values across each of the four models under the optimum sampling scheme for $n = 2,600$. Plotting symbols indicate village numbers.

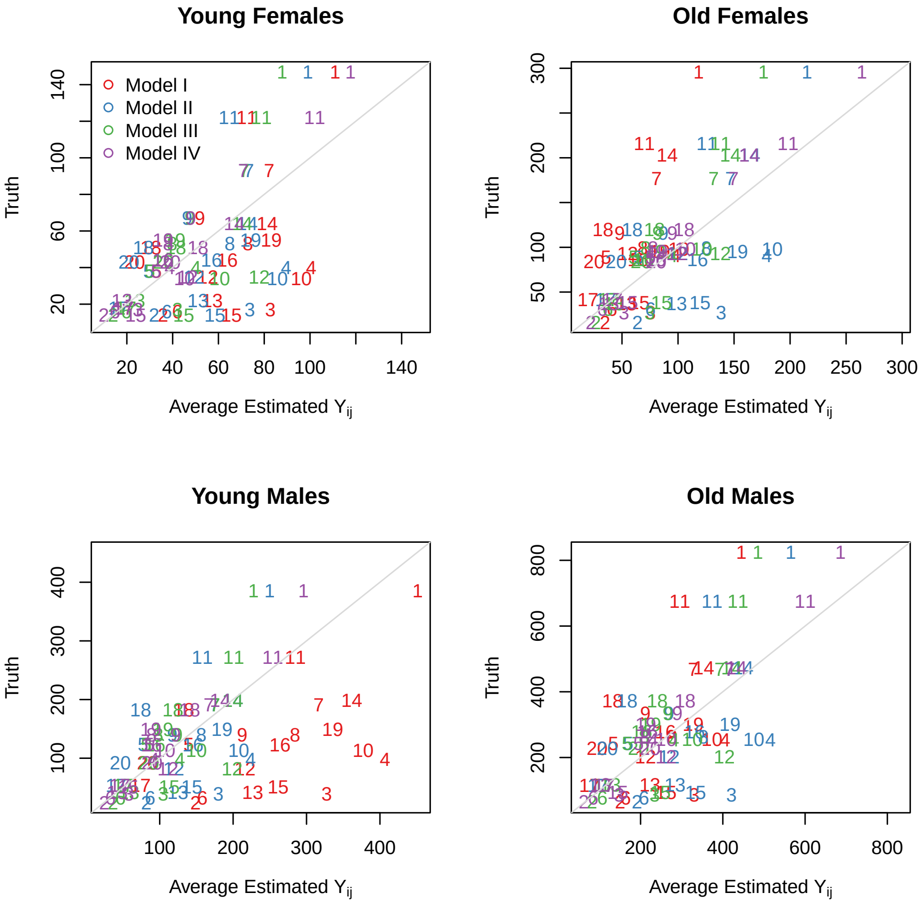

Figure A.18: The average village- and strata-specific estimates for the (unobserved) population counts of death plotted against the true values across each of the four models under the optimum sampling scheme for $n = 1,300$. Plotting symbols indicate village numbers.
